# Lung Clearance Index in Children with Cystic Fibrosis during Pulmonary Exacerbation

**DOI:** 10.3390/jcm10214884

**Published:** 2021-10-23

**Authors:** Katarzyna Walicka-Serzysko, Magdalena Postek, Justyna Milczewska, Dorota Sands

**Affiliations:** 1 Cystic Fibrosis Department, Institute of Mother and Child, 01-211 Warsaw, Poland; magdalena.postek@imid.med.pl (M.P.); justyna.milczewska@szpitaldziekanow.pl (J.M.); dorota.sands@imid.med.pl (D.S.); 2 Cystic Fibrosis Centre, Pediatric Hospital, 05-092 Warsaw, Poland

**Keywords:** cystic fibrosis, lung clearance index, pulmonary exacerbation

## Abstract

(1) Background: Pulmonary exacerbation (PEx) is one of the main factors affecting the quality of life and life expectancy in patients with cystic fibrosis (CF). Our study aimed to evaluate the change in selected pulmonary function parameters, including lung clearance index (LCI), in patients with CF diagnosed with PEx. (2) Methods: We enrolled 40 children with CF aged 6–17. They performed spirometry and multiple breath nitrogen washout (MBNW) tests during a stable condition period at the beginning and the end of intravenous antibiotic treatment. (3) Results: LCI increased by 65% and FEV_1_ decreased by ≥10% in 40% of patients with CF during PEx. An absolute change in LCI between a stable condition period and PEx was 1.05 (±1.92) units, which corresponds to a relative change of 11.48% (±18.61) of the baseline. The relative decrease in FEV_1_ was −9.22% (±12.00) and the z-score was −0.67 (±1.13). After the PEx treatment, FEV_1_ increased by 11.05% (±9.04) on average, whereas LCI decreased by 1.21 ± 1.59 units on average, which represented 9.42% ± 11.40 compared to the value at the beginning of PEx. (4) Conclusions: The change in LCI captures a higher proportion of events with functional impairment than FEV_1_ in school-age children with CF.

## 1. Introduction

Advances in the clinical care of patients with cystic fibrosis (CF) in the past six decades have significantly improved health outcomes [1]. The natural history of CF lung disease begins immediately after birth and gradually progresses with acute episodes of worsening respiratory symptoms through childhood into adulthood. The enhancement in the multidisciplinary care and development of new therapies, such as CFTR modulators, have changed the natural trajectory of the disease [1]. This leads to the attenuation of lung damage and maintenance of spirometry lung function within the normal range from childhood into early adulthood.

However, pulmonary exacerbation (PEx) is still one of the main factors affecting the quality of life and life expectancy of individuals with CF. It remains an important clinical event in the course of cystic fibrosis that increases the risk of lung transplant and mortality [2].

Pulmonary function measurements are well established in evaluating advanced lung disease in adolescent and adult patients. In particular, forced expiratory volume in 1 s (FEV_1_) is the main parameter used in assessing and monitoring CF lung disease. It is also used for diagnosing and detecting treatment response of PEx [3,4]. Nevertheless, improvements in lung function have decreased the sensitivity of spirometry, making it difficult to evaluate and track early disease progression. This observation emphasizes the need to search for tools that can provide more sensitive results [5]. Therefore, new markers of lung function correlating with structural changes are being sought and investigated.

The lung clearance index (LCI), which is derived from multiple breath nitrogen washout (MBNW), is a sensitive parameter in early CF lung disease and is increasingly used to assess lung function in stable condition periods as well as during PEx [6,7,8,9]. It might be an alternative and more useful indicator than FEV_1_ to estimate treatment response in the growing number of children and young adults with CF who have spirometry within the normal range. Early CF lung disease usually arises peripherally, whereas FEV_1_ allows us to detect changes in the large airways. Therefore, LCI is a more sensitive marker than spirometry in detecting early signs of lung disease [10,11]. It is repeatable, and its results correlate with high-resolution computed tomography outcomes [12]. Due to an attractive feasibility and clinimetric properties profile, it is particularly indicated for multicenter trials in young children with CF and patients with early or mild CF lung disease [13,14]. LCI might also be a more sensitive option than FEV_1_ in detecting response to intervention in patients with mild lung disease. It has now been chosen as the main outcome measure in clinical trials with CFTR modulators in young people with CF [15].

Therefore, this parameter is increasingly used in clinical practice to gauge lung function in stable periods as well as during PEx in CF [12,16,17,18].

Reference values for LCI and other MBNW outcomes were established in Caucasian healthy school-aged children [19]. However, there are scant data regarding the usefulness of LCI to monitor the evolution of ventilation distribution during and after PEx treatment in children with CF [9,16,20,21]. A percentage change in LCI greater than ±15% in preschool and school-aged children can be considered physiologically relevant [6,7,22]. Furthermore, in contrast to studies where FEV_1_ was used as the primary outcome, which consistently showed a positive treatment effect [23,24,25], studies evaluating the ability of LCI to perceive treatment response for PEx have given heterogeneous results [12,16,20,26,27,28].

The aim of our study was to evaluate the change in LCI in comparison with FEV_1_ in children with CF diagnosed with PEx requiring intravenous antibiotic therapy. This trial was presented online in virtual poster form at the 34th Annual North American Cystic Fibrosis Conference, 7–23 October 2020.

## 2. Materials and Methods

### 2.1. Study Design

This retrospective observational survey was carried out at the Polish Cystic Fibrosis Centre from February 2017 to September 2019. The study was conducted according to the principles outlined in the Declaration of Helsinki. The protocol was approved by the local ethics committee at the Institute of Mother and Child in Warsaw (opinion number 4/2019). After obtaining approval from the local ethics committee, the participants and caregivers gave their informed consent for the use of their test results (microbiological examinations, respiratory function tests) in the study.

Consecutive pediatric patients with CF admitted to the hospital for intravenous antibiotic therapy due to PEx underwent a clinical assessment in order to determine their eligibility. Subjects who met all of the inclusion criteria (male or female aged 6–17, a confirmed diagnosis of CF based on current diagnostic criteria [29,30], ability to perform lung function tests: MBNW and spirometry) and none of the exclusion criteria (lack of cooperation, severe clinical condition precluding a patient from performing MBNW and spirometry, e.g., hemoptysis, pneumothorax, and other severe complications of CF) were included in the trial.

PEx was defined as an acute worsening of respiratory symptoms (e.g., increased coughing and sputum production, change in sputum and shortness of breath), systemic symptoms (e.g., fever, weight loss, and fatigue), and new clinical findings (such as new crackles or wheezing, decreased respiratory function in tests, and new radiological findings) according to Fuchs criteria [31]. The local standards of admission for intravenous antibiotic therapy is failure of oral treatment or severe clinical status during PEx. The following variables were collected: age, sex, height, weight, body mass index, and intravenous antibiotic treatment duration. The lung function tests: MBNW and spirometry were carried out at the start (±48 h) and the end (±24 h) of intravenous antibiotic therapy.

Microbiological samples: sputum or throat swab were collected at the admission to hospital and cultured for various bacterial species, including *Staphylococcus aureus* (methicillin-sensitive and methicillin-resistant strains), *Haemophilus influenzae*, *Stenotrophomonas maltophilia, Pseudomonas aeruginosa, Burkholderia cepacia* as well as fungal species, including *Aspergillus fumigatus*. Infection status was based on the current results compared to outcomes obtained in the previous year.

Based on the medical documentation of routine visits at our center, the clinical data (MBNW, spirometry, and culture results) concerning the stable baseline 3–6 months prior to the exacerbation were also analyzed. A stable condition was defined as no symptoms or signs of PEx according to Fuchs criteria [31]. Data regarding genotype, pancreatic status (PS), CF comorbidities such as cystic-fibrosis-related diabetes (CFRD), and allergic bronchopulmonary aspergillosis (ABPA) were obtained from the medical records.

### 2.2. Pulmonary Function Measurements

Subjects with CF performed an MBNW and spirometry test after having completed their standard daily airway clearance therapy. In order to avoid the effect of forced breathing for the results of the MBNW test, it was carried out before spirometry. All lung function measurements were performed in accordance with the standard European Respiratory Society (ERS) guidelines for lung function measurements. The technical correctness of the tests was checked by taking into account the guidelines provided by Jensen et al. [32].

Exhalyzer-D (EcoMedics AG, Duernten, Switzerland, software version 3.2.0) was used to conduct the MBNW tests. The session was approved if there were at least two or more technically acceptable trials according to guidelines in the ERS/ATS consensus statement [33]. All trials were assessed for breathing pattern in order to ensure it was representative of relaxed tidal breathing with no evidence of hyperventilation or leaks. The MBNW results are presented as the mean (SD) of three technically acceptable results. Correctness of maneuvers was determined by the software and also by the operator’s assessment who observed and controlled the patient’s cooperation.

The Jaeger Vyntus IOS (CareFusion, Hochberg, Germany) was used to perform spirometry. All tests were carried out in accordance with the American Thoracic Society/European Respiratory Society criteria [34,35].

All instruments were calibrated on the day of the examination. The spirometer was calibrated with a 3 L syringe. The error of measuring the linearity and repeatability did not exceed ±2.5%. Temperature and barometric pressure, saturated with water vapor, were updated daily before measurements. Reference values for children and adolescents were used [36,37].

### 2.3. Statistical Analysis

All values, expressed as z-scores, were based on gender- and age-specific regression equations. The normality of the data distributions was determined by the Shapiro–Wilk test and graphical analysis. The homogeneity of variance was examined using the Brown–Forsythe test. In the analysis of paired data with a normal distribution, the Student’s *t*-test was used. In cases of paired data in which the distribution was not normal, the Wilcoxon test was used. The Spearman correlation was used to determine the correlation between the values. Data were analyzed with STATISTICA version 13.3.

The relative change in LCI and FEV_1_ was calculated according to the following formulas:

((LCI_PEx_ − LCI_baseline_)/LCI_baseline_) × 100%

(FEV_1_% pred _PEx_ − FEV_1_% pred _baseline_)

## 3. Results

### 3.1. Subject Characteristics

Over the 32 months of the recruitment period, 40 patients with CF aged 6–17 (14 males; 26 females) were enrolled in the trial. The baseline characteristics of the study cohort fulfilling the inclusion criteria are presented in Table 1. The average length of antibiotic therapy was 13 days ± 1 day [10,11,12,13,14,15,16,17]. Amikacin and ceftazidime were the most commonly used antibiotics for treatment (65%), colistin and ceftazidime were used in 5%, and other therapy in 30% of patients.

### 3.2. Pulmonary Function Measurements

#### 3.2.1. Change in LCI and FEV_1_ at the Beginning of PEx

**Figure 1:**
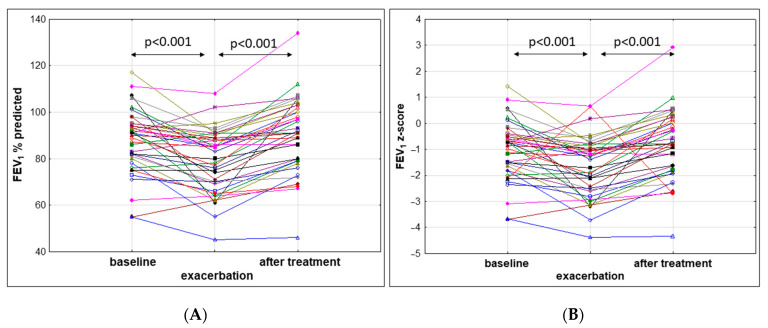
FEV_1_ results: (**A**) FEV_1_ % predicted and (**B**) FEV_1_ z-score for subjects with CF in stable condition, before and after treatment of pulmonary exacerbation.

Thirty-six subjects (90%) had mild lung disease (FEV_1_ > 80% pred) and four (10%) had moderate lung disease (40% pred ≤ FEV_1_ ≤ 80% pred). The median FEV_1_ at admission was 79.48 ± 13.19% pred. The relative decrease in FEV_1_ at the beginning of PEx was −9.22% (±12.00; CI 95%: 9.83–15.42) and the z-score was −0.67 (±1.13). The decline in FEV_1_ by ≥ 10% was observed in 16 (40%) patients (Figure 1).

**Figure 2:**
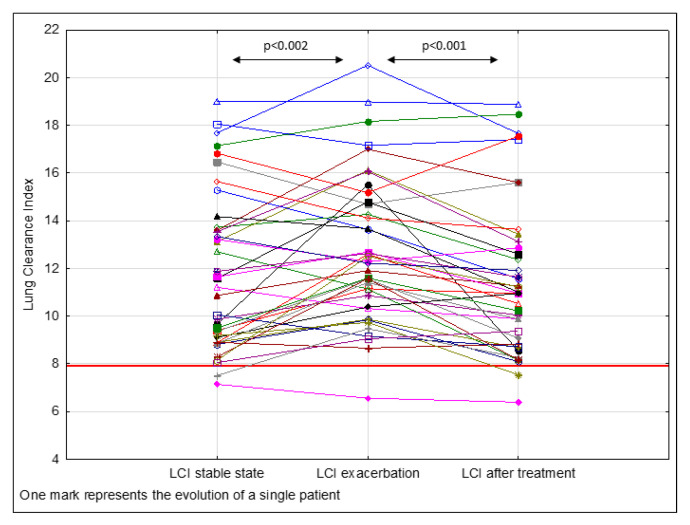
Lung clearance index (LCI) values for CF subjects in stable condition, before and after treatment of pulmonary exacerbation. For LCI, the upper limit of 7.91 was presented according to normative data for healthy children [19].

Under stable conditions, LCI ≥ 7.91 was reported in 37 (92.5%) patients, the median was 11.04 [8.9–13.7]. Upon admission, the mean (SD) LCI was 12.70 (±3.10) units (Figure 2). An increase in LCI values was reported in 65% of CF patients (*n* = 26) during PEx. A relative change in LCI greater than 5%, 10%, and 15% was observed in 62.5%, 50%, and 37.5% subjects, respectively. An absolute change in LCI between a stable condition period and exacerbation was 1.05 (±1.92) units, which corresponds to a relative change of 11.48% (±18.61; CI 95%: 5.53–17.43) from the baseline. LCI response was not uniform in all patients. In 14 subjects (35%), we observed a decrease in LCI. In patients with a decrease in FEV_1_%pred <5%, we observed an increase in LCI of 4.78 ± 14.20% [−9.79–27.52%], in patients with 5–9.99% decrease, it was 4.69 ± 18.22% [−10.79–39.67%], and in patients with ≥10% decrease, 19.23 ± 21.04% [−12.28–60.66%].

#### 3.2.2. Pulmonary Function Response to PEx Treatment

**Figure 3:**
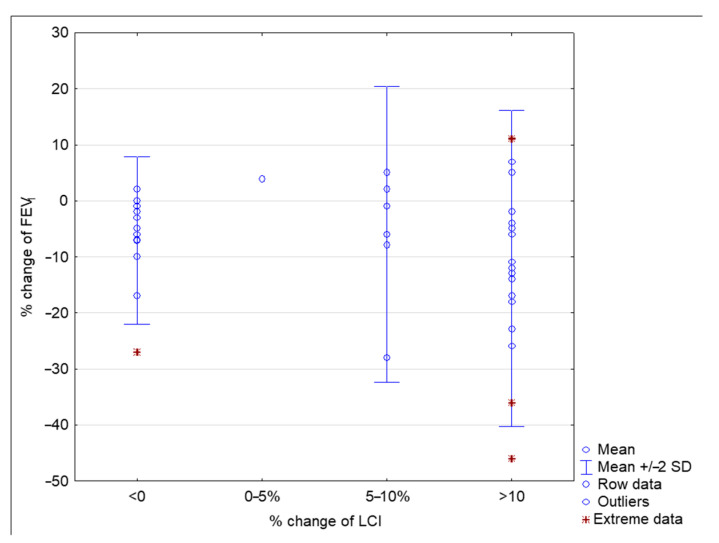
FEV_1_ and LCI change between the start and end of PEx treatment.

After antibiotic therapy, overall LCI decreased significantly (*p* < 0.001) by 1.21 ± 1.59 units on average, which represented 9.42% ± 11.40 compared to the value at the beginning of PEx. Spirometry parameters FEV_1_ and FVC increased from 79.48 ± 13.19% pred. to 90.53 ± 15.44% pred. (*p* < 0.001), and from 92.98 ± 13.02% pred. to 98.68 ± 13.26, respectively (*p* < 0.001). After the PEx treatment, FEV_1_ increased by 11.05% (±9.04) on average. FEV_1_ and LCI changes between the start and end of PEx treatment are presented in Figure 3. Changes in FEV_1_, FVC, and LCI during the stable condition, PEx, and after antibiotic therapy for the study population are outlined in Table 2.

## 4. Discussion

PEx in the course of CF is a well-characterized, significant factor, which has negative consequences on clinical outcomes [2,38]. Each PEx occurrence poses a risk of permanent lung function decline [25]. It was confirmed that approximately 25% of patients did not recover to the FEV_1_ baseline at the end of treatment [23]. The frequency of PEx is closely associated with a subsequent reduction in lung function [3,39,40,41].

This study was conducted to evaluate the usefulness of LCI in diagnosing and detecting treatment response in children with cystic fibrosis who required intravenous antibiotic therapy for PEx. In 40 patients between 6 and 17 years of age enrolled in the trial, selected pulmonary function parameters obtained from the MBNW and spirometry tests were compared. The longitudinal courses of FEV_1_ and LCI were assessed on three-time points: stable, hospital admission for PEx, and after intravenous antibiotic treatment. The study results demonstrate that a ≥10% increase in LCI captures a higher proportion of events with functional impairment than FEV_1_ in school-age children with CF. Both of these parameters relate to different aspects of lung physiology. LCI is more sensitive to changes in the small airways where early lung disease begins. In turn, FEV_1_ is associated with changes in central airways during CF in patients with advanced lung disease. According to previous studies, a percentage increase in LCI greater than 15% is clinically relevant in preschool and school-aged children [6,7,22]. In our opinion, a change in LCI in the range of 10% to 15% can be considered clinically meaningful and could be used to make a clinical decision.

For decades, the technique of choice for monitoring respiratory status in CF patients was conventional spirometry. Nowadays, in the new era of CF care with the introduction of CFTR modulators, the progression of lung disease has slowed down [41] and FEV_1_ has become a less sensitive outcome measure.

In our study, based on spirometry performed during a stable period, most subjects (*n* = 36; 90%) had mild lung disease (FEV_1_ > 80% pred) and four (10%) had moderate lung disease (40% pred ≤ FEV_1_ ≤ 80% pred). An exacerbation was diagnosed based on Fuchs criteria. A decrease in pulmonary function (FEV_1_) by 10% or more from a previously recorded value was observed in only 16 (40%) subjects. Most of the children did not fulfill Fuchs criteria regarding spirometry outcomes. The relative decrease in FEV_1_ during exacerbation was −9.22% (±12.00) and the z-score was −0.67 (±1.13). After the treatment of PEx, FEV_1_ increased by 11.05% (±9.04) on average. This observation confirms that in children with early lung disease, spirometry is not sensitive enough to diagnose PEx.

Maintaining FEV_1_ values within normal ranges in subjects with early lung disease or younger patients who are unable to properly perform spirometry tests has led to the wider use of the MBNW technique in monitoring lung function in the course of cystic fibrosis [4,5].

According to the new reference values reported for MBNW outcomes measured on the same equipment (Exhalyzer D, Eco Medics AG), for healthy Caucasian school-aged children, the ULN for LCI is 7.91. In our study, most of the patients (*n* = 37; 92.5%) in stable condition had LCI > 7.91 and only 3 (7.5%) subjects had LCI < 7.91.

LCI is a sensitive and reproducible marker of ventilation inhomogeneity [13]. However, knowledge of LCI variation and fluctuation over time is crucial for understanding when changes in ventilation inhomogeneity are clinically meaningful. In our study, we noticed an increase in LCI in 65% of CF patients during PEx. The relative change in LCI between a stable condition period and exacerbation was 1.05 (±1.92) units, which corresponds to a relative change of 11.48% (±18.61; CI 95%: 5.53–17.43) from the baseline.

Observations in healthy children and adolescents show that acceptable short and long variability of LCI measurements ranging from 4.2% to 5.1% for one- and six month-intervals respectively. Furthermore, in the healthy spectrum of LCI, an absolute change of approximately one unit indicates a clinically relevant change [42]. Similar results were obtained by Singer et al. in a study with CF children and controls. It showed that the coefficient of repeatability between tests occasions performed 24 h apart was 0.96 in CF children compared to 0.62 in controls. This observation also shows that a change in LCI of at least one unit is clinically meaningful in children with CF [10].

In our study, increases in LCI greater than 5% were noted in 62.5% of children with PEx. Half of the patients had a relative increase in LCI greater than 10%, and 15 patients (37.5%) more than 15%, respectively. A change in LCI as an increase of 10% or more from baseline was the better indicator of PEx than FEV_1_. Clinical research conducted over the past few years has demonstrated that changes in LCI beyond 15% are clinically significant in children with cystic fibrosis [6,7,22]. However, the 10% threshold may facilitate the diagnosis of respiratory events in clinical practice.

When comparing LCI during the stable condition and start of antibiotic therapy in 14 subjects (35%), we observed a decrease in this parameter. Some hypotheses could explain the heterogeneity of the LCI results. The overall heterogeneity of ventilation on the LCI will be influenced by the persistent abnormalities of the respiratory tract caused by fibrotic and destructive processes and modifiable differences in inflammation and mucus retention escalating, especially during PEx [12,26]. These abnormalities, such as mucus plugging, could entirely block airways that were partially ventilated in stable conditions, causing some of them to become non-ventilating and contribute to the decrease in overall heterogeneity, and thus, LCI during exacerbation.

LCI as a sensitive marker of ventilation heterogeneity is increasingly used for the detection of treatment effects of variable interventions [15,43]. However, the LCI response to antibiotic therapy for PEx has yielded heterogeneous results. In a systematic review, there was a retrospective analysis of pooled LCI data conducted in order to assess the efficacy of therapeutic interventions and to understand factors explaining the heterogeneous response [16]. Seven studies of 176 pulmonary exacerbations in both pediatric and adult patients were included in the survey. Overall, LCI decreased significantly by 0.40 units, or 2.5%, following treatment. A significant but not necessarily clinically relevant effect of antibiotic treatment was recorded. In another study, a paradoxical increase in LCI was observed after PEx treatment [17]. During our trial, we found that after the PEx treatment, LCI decreased by 1.21 ± 1.59 units on average, which represented 9.42% ± 11.40 compared to the value at the beginning of the exacerbation.

Rayment et al. investigated pulmonary function parameters to monitor treatment response in PEx in preschool CF children. They analyzed LCI changes during lower respiratory tract symptoms relative to a recent clinically stable measurement. In this study, LCI increased from baseline in both treated (mean relative change +23.8%) and untreated symptomatic visits (mean relative change +11.2%). A significant antibiotic treatment effect was noticed when LCI was used as the outcome measure (average treatment effect −15.5%) but not for the z-score FEV_1_ [9].

Our study, as well as those mentioned in the Discussion, indicate the need for further research into LCI use in diagnosing PEx in children with cystic fibrosis. This sensitive parameter could be useful in the diagnosis of PEx and in monitoring children with early lung disease during routine clinical surveillance. The MBNW technique could be helpful especially in young patients who often cannot perform spirometry and adolescents with mild lung disease. Longitudinal analysis is needed to assess LCI potential as a criterion of PEx diagnosis in children with CF and as an outcome measure in detecting treatment response.

It is very important to integrate MBNW into routine practice with patients with early lung disease. LCI seems to be a good parameter that can help clinicians diagnose PEx in children with cystic fibrosis and monitor response to the treatment.

From the authors’ perspective, LCI is likely to become a valuable tool in clinics especially in children with preserved lung function to identify PEx. In young children (3–5 years old) who cannot perform spirometry, MBNW is the only lung function test that can help in recognizing PEx. In older children, this technique has still been a complementary tool to spirometry. However, there remain some limitations for wider use, particularly the skills and time it takes to obtain reliable maneuvers in children and particularly in those younger than 6 years of age. We are aware that a small group of children with cystic fibrosis were enrolled in our study. Therefore, we cannot generalize results to the whole population. We think that the recommended cut-off for LCI relative change should be 15%. However, an increase above 11% may indicate the need for careful observation and clinical decision-making, such as improving physiotherapy, checking adherence, or starting antibiotic therapy. This study has several methodological limitations. The overall sample size is limited. Outcomes were based on unstandardized clinical treatment decisions (e.g., antibiotic choice and duration). The treatment effect observed in pulmonary function parameters was attributed to antibiotic therapy, but treatment for PEx will also often include the modification of inhaled medications and aggressive chest physiotherapy. These factors could have contributed to the observed treatment effect. The study is focused on school children; however, this narrow group characteristic may not be generalizable to the entire CF population.

## 5. Conclusions

In the era of better care of patients with cystic fibrosis, there is increasing interest in using the MBNW technique for detecting early changes in lung disease.

Our study results suggest that the change in LCI captures a higher proportion of events with functional impairment than FEV_1_ in school-age children with CF during PEx. As FEV_1_ changed significantly during PEx, compared to baseline, and improved with antibiotic therapy, LCI shows a greater promise in clinical practice, particularly in younger patients with mild disease.

As we gain more insight into how changes in LCI can be interpreted to guide clinical decisions, MBNW testing may become an integral part of the clinical management of children with cystic fibrosis.

## Figures and Tables

**Table 1:** Characteristics of the study population and its microbiological status (BMI = body mass index, CFRD = cystic fibrosis-related diabetes, ABPA = allergic bronchopulmonary aspergillosis, MSSA = methicillin-sensitive *Staphylococcus aureus,* MRSA = methicillin-resistant *Staphylococcus aureus*).

Characteristics	
Patients, *n* (%)	40 (100%)
Females, *n* (%)	26 (65%)
Age at admission (years)	12.7 ± 2.86
Height (meters)	1.55 ± 0.14
Height *z*-score	0.10 ± 1.09
Weight (kg)	45.06 ± 11.46
Weight *z*-score	−0.15 ± 0.97
BMI	18.29 ± 2.12
BMI *z*-score	−0.21 ± 0.77
*CFTR* genotype	
F508del/F508del, *n* (%)	14 (35%)
F508del/other, *n* (%)	19 (47.5%)
other/other, *n* (%)	7 (17.5%)
CF comorbidities	
Pancreatic insufficiency, *n* (%)	35 (87.5%)
CFRD, *n* (%)	7 (18%)
ABPA, *n* (%)	1 (2.5%)
Microbiology	
*P. aeruginosa*, *n* (%)	23 (57.5)
-chronic, *n* (%)	12 (52)
-intermittent, *n* (%)	6 (26)
-first time, *n* (%)	5 (22)
MSSA, *n* (%)	35 (87.5)
MRSA, *n* (%)	1 (2.5)
*H. influenzae*, *n* (%)	5 (12.5)
*S. maltophilia*, *n* (%)	3 (7.5)
*B. cepacia complex*, *n* (%)	0
*A. fumigatus*, *n* (%)	6 (15)

**Table 2:** Lung clearance index (LCI), forced expiratory volume in 1 s (FEV_1_), and forced vital capacity (FVC) values during the stable condition, at hospital admission, and after pulmonary exacerbation (PEx) treatment for the study population (*n* = 40 patients).

Parameter Average (SD)	Baseline	P Baseline vs. Admission to Hospital	At Admission to Hospital	P Exacerbation vs. after Treatment	After PEX Treatment
FVC %pred	97.80 ± 12.41	<0.001	92.98 ± 13.02	<0.001	98.68 ± 13.26
FVC *z*-score	−0.20 ± 1.07	<0.001	−0.61 ± 1.11	<0.001	−0.12 ± 1.13
FEV_1_%pred	88.70 ± 14.26	<0.001	79.48 ± 13.19	<0.001	90.53 ± 15.44
FEV_1_ *z*-score	−0.95 ± 1.18	<0.001	−1.62 ± 1.12	<0.001	−0.79 ± 1.28
LCI	11.65 ± 3.34	<0.002	12.70 ± 3.10	<0.001	11.40 ± 3.22

## Data Availability

The data presented in this study can be provided by the corresponding author on reader’s request.
